# Assessment of Oral Fluid HIV Test Performance in an HIV Pre-Exposure Prophylaxis Trial in Bangkok, Thailand

**DOI:** 10.1371/journal.pone.0145859

**Published:** 2015-12-30

**Authors:** Pravan Suntharasamai, Michael Martin, Kachit Choopanya, Suphak Vanichseni, Udomsak Sangkum, Pairote Tararut, Wanna Leelawiwat, Rapeepan Anekvorapong, Philip A. Mock, Thitima Cherdtrakulkiat, Manoj Leethochawalit, Sithisat Chiamwongpaet, Roman J. Gvetadze, Janet M. McNicholl, Lynn A. Paxton, Somyot Kittimunkong, Marcel E. Curlin

**Affiliations:** 1 Bangkok Tenofovir Study Group, Bangkok, Thailand; 2 Thailand MOPH – U.S. CDC Collaboration, Nonthaburi, Thailand; 3 Centers for Disease Control and Prevention, Atlanta, Georgia, United States of America; 4 Bangkok Metropolitan Administration, Bangkok, Thailand; 5 Thailand Ministry of Public Health, Nonthaburi, Thailand; University of Athens, Medical School, GREECE

## Abstract

**Background:**

Rapid easy-to-use HIV tests offer opportunities to increase HIV testing among populations at risk of infection. We used the OraQuick Rapid HIV-1/2 antibody test (OraQuick) in the Bangkok Tenofovir Study, an HIV pre-exposure prophylaxis trial among people who inject drugs.

**Methods:**

The Bangkok Tenofovir Study was a randomized, double-blind, placebo-controlled trial. We tested participants’ oral fluid for HIV using OraQuick monthly and blood using a nucleic-acid amplification test (NAAT) every 3 months. We used Kaplan-Meier methods to estimate the duration from a positive HIV NAAT until the mid-point between the last non-reactive and first reactive oral fluid test and proportional hazards to examine factors associated with the time until the test was reactive.

**Results:**

We screened 3678 people for HIV using OraQuick. Among 447 with reactive results, 436 (97.5%) were confirmed HIV-infected, 10 (2.2%) HIV-uninfected, and one (0.2%) had indeterminate results. Two participants with non-reactive OraQuick results were, in fact, HIV-infected at screening yielding 99.5% sensitivity, 99.7% specificity, a 97.8% positive predictive value, and a 99.9% negative predictive value. Participants receiving tenofovir took longer to develop a reactive OraQuick (191.8 days) than participants receiving placebo (16.8 days) (p = 0.02) and participants infected with HIV CRF01_AE developed a reactive OraQuick earlier than participants infected with other subtypes (p = 0.04).

**Discussion:**

The oral fluid HIV test performed well at screening, suggesting it can be used when rapid results and non-invasive tools are preferred. However, participants receiving tenofovir took longer to develop a reactive oral fluid test result than those receiving placebo. Thus, among people using pre-exposure prophylaxis, a blood-based HIV test may be an appropriate choice.

**Trial Registration:**

ClinicalTrials.gov NCT00119106.

## Introduction

Globally, half of those infected with HIV do not know their status.[1] These individuals do not have access to life-saving antiretroviral therapy and may unknowingly transmit HIV to others. In addition, people often learn they are infected with HIV late in their illness.[2] In response, UNAIDS has urged countries to ensure that by 2020, 90% of people infected with HIV know their status,[3] and the World Health Organization has called for national HIV programs to identify people living with HIV as early as possible and link them to HIV services in a timely manner.[4]

Rapid HIV tests have created opportunities to increase testing by providing results the same day, often within 30 minutes, and by extending testing beyond medical facilities.[5] In 2004, the U.S. Food and Drug Administration approved the OraQuick Advance Rapid HIV-1/2 Antibody Test (OraSure Technologies, Inc., Bethlehem, Pennsylvania, USA) for use with oral fluid, and in 2012 it became the first over-the-counter HIV test approved for home use.[6] Oral fluid HIV tests have not been approved to diagnose HIV in Thailand.[7,8]

We used an oral fluid HIV test in the Bangkok Tenofovir Study, an HIV pre-exposure prophylaxis trial among people who inject drugs.[9] We chose the test because it could be done in drug treatment clinics, provided a result in 20 minutes, did not require a blood draw, and had good reported sensitivity and specificity.[10] Here, we describe the performance of the test.

## Methods

The Bangkok Tenofovir Study was a randomized, double-blind, placebo-controlled trial conducted in 17 drug-treatment clinics that showed that daily oral tenofovir disoproxil fumarate (tenofovir) can reduce HIV transmission among people who inject drugs by 49%.[9] The study protocol, consent process, and trial materials were approved by the Bangkok Metropolitan Administration and Thailand Ministry of Public Health Ethical Review Committees and the U.S. Centers for Disease Control and Prevention (CDC) Institutional Review Board. An independent Data and Safety Monitoring Board conducted annual safety reviews and one interim efficacy review. Participants provided written informed consent to participant in the study. The trial was registered with ClinicalTrials.gov Identifier: NCT00119106.

### Participants and procedures

HIV-uninfected people who met eligibility criteria and provided written informed consent were randomly assigned to receive daily oral tenofovir 300 mg or placebo.[11] Participant screening and enrollment began in June 2005 and follow-up continued through October 2012. At screening, enrollment, and each monthly visit, staff observed participants collect an oral fluid specimen and tested the specimen using OraQuick Rapid HIV-1/2 Antibody Test (OraQuick).[12] OraQuick is manufactured for distribution outside the United States and is identical to the U.S. Food and Drug Administration approved OraQuick Advance Rapid HIV-1/2 Antibody test.[10] We confirmed reactive oral fluid HIV tests using enzyme-immunoassays (EIA) (Genetic Systems HIV-1/ HIV-2 plus O EIA, Redmond, WA, USA) and Western blot (Bio-Rad, Redmond, WA, USA).

We collected blood specimens from participants at enrollment, months 1, 2, 3, and every 3 months thereafter, for safety assessments. Blood collected at 3-monthly visits from participants with non-reactive oral fluid HIV test results was tested for HIV using EIA. We tested the final blood specimens from participants with consistently non-reactive results for HIV using nucleic-acid amplification testing (NAAT) (Aptima HIV-1 RNA Qualitative Assay, GenProbe Inc, San Diego, CA, USA). Samples with positive NAAT results were confirmed and viral load determined using real-time polymerase chain reaction (PCR) (COBAS TaqMan v 1.0, Roche Molecular Systems, Branchburg, NJ, USA). We determined the last NAAT negative and first NAAT positive specimen using real-time PCR. The lower limit of detection was 47 copies/mL. We used TruGene (Siemens HealthCare Diagnostic, Tarrytown, NY, USA) to determine HIV subtype.

OraSure personnel trained CDC staff on the biological principles of the OraQuick test, kit storage, specimen collection, testing and interpretation, and quality control.[10] CDC staff trained clinic-based staff who performed participant testing. After training, performance was assessed using proficiency panels from the Model Performance Evaluation Program and the Thailand National Institute of Health. CDC staff visited each clinic monthly to review HIV testing records and observe staff performing tests.

### Statistical analysis

If the HIV NAAT was positive before the oral fluid test, we estimated the time until the oral fluid test was reactive using the date of the first positive NAAT and the mid-point date between the last non-reactive and first reactive oral fluid test. We used the Kaplan-Meier method to estimate the time from a positive HIV NAAT until a reactive oral fluid test result, using the mid-point date, and a Cox proportional hazards model, including study drug group, to determine factors associated with the time until the test was reactive using the likelihood ratio chi-square test.[13] We compared HIV RNA concentrations when the HIV NAAT was positive by study drug group using a non-parametric Wilcoxon test; to assess RNA concentration by other factors, we adjusted for study drug group using van Elteren’s test.[13,14] We excluded data from participants lost to follow-up for more than 3 months who had a positive NAAT when they returned, and censored data of participants who did not have a reactive oral fluid HIV test the date of their final test. We used SAS version 9.3 (SAS Institute, Cary, North Carolina, USA) to analyze data.

## Results

**Fig 1:**
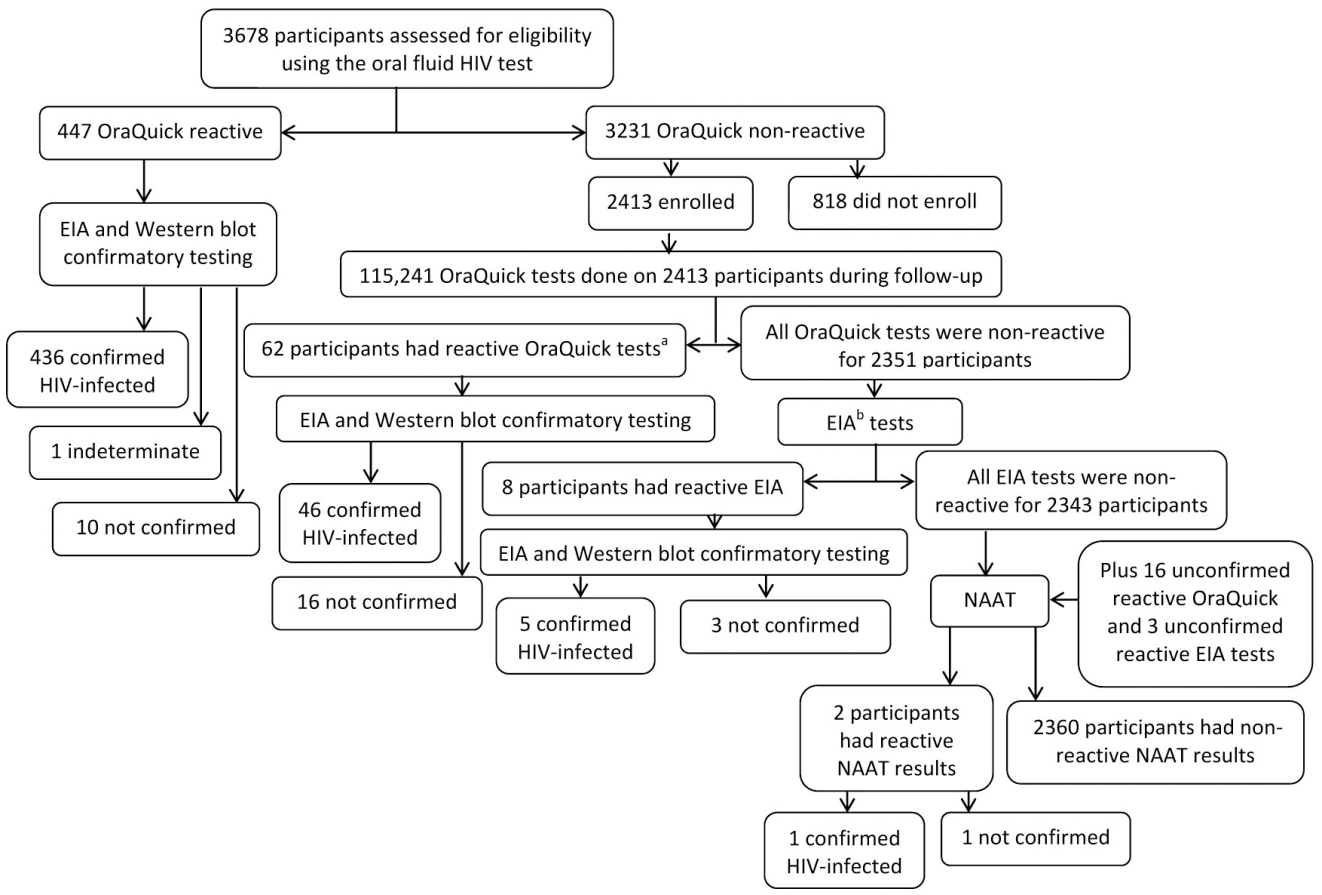
HIV test results of participants in the Bangkok Tenofovir Study, 2005–2012. OraQuick, OraQuick Rapid HIV-1/2 Antibody Test; EIA, enzyme immune assay; NAAT, nucleic-acid amplification test. ^a^Two participants with reactive OraQuick tests during follow-up were later found to have been HIV-infected before enrollment. ^b^Plasma collected at 3-monthly visits from participants with a non-reactive OraQuick test result was tested for HIV using EIA.

From June 2005 through July 2010, 3678 people were screened using the oral fluid OraQuick HIV test. Among those screened, 447 participants had a reactive result: 436 (97.5%) were confirmed HIV-infected, 10 (2.2%) had a falsely reactive result, and one (0.2%) had an indeterminate Western blot result and was excluded from analysis (Fig 1). Two participants with non-reactive oral fluid HIV test results were later found to have been HIV-infected before enrollment, based on blood EIA and Western blot testing. These results yielded a sensitivity of 99.5% (95% confidence interval [CI]: 90.4% to 100%), a specificity of 99.7% (95% CI: 96.3% to 100%), a positive predictive value of 97.8% (95% CI: 88.8% to 100%), and a negative predictive value of 99.9% (95% CI: 96.5% to 100%).

Among those screened, 2413 enrolled. Their median age was 31 years (range, 20–59), and 1924 (79.7%) were male. Enrolled participants had 115,241 oral fluid HIV tests done from June 2005 through June 2012, ranging from 1 to 88 tests per participant, and from 4079 to 10,973 tests per clinic.

Among those enrolled, 62 participants had a reactive oral fluid HIV test result during follow-up; 46 (74.2%) participants were confirmed HIV-infected and 16 (25.8%) had falsely reactive results. Two of the confirmed HIV-infected participants were later found to have been infected before enrollment (described above) and excluded from analysis (Fig 1). The 16 falsely reactive results were reported from nine clinics and occurred between June 2006 and February 2012 during study visits ranging from the first monthly visit to the month 81 visit. Five participants were diagnosed with HIV infection using EIA and Western Blot testing and did not have a reactive oral fluid HIV test result reported. Final blood specimens collected from two participants tested positive for HIV using NAAT; one was confirmed, while the other could not be confirmed because there was insufficient blood and the participant could not be reached for repeat testing (Fig 1).

Among the 50 participants with confirmed incident HIV infection, 10 were lost to follow-up for more than 3 months and returned HIV-infected. We excluded these participants from further analysis. Of the 40 remaining participants, 14 (35.0%) had a reactive oral fluid HIV test the same day the NAAT was reactive, and, including these 14, 27 (67.5%) participants had reactive oral fluid HIV test results within 3 months (i.e., 84 days) of the positive NAAT. Staff reported reactive test results for 8 (20.0%) participants more than 3 months after the positive NAAT. The eight participants came from six clinics, and the first positive NAAT results occurred between March 2006 and June 2010 and on study visits ranging from month 9 to month 54. In addition, 5 (11.9%) participants with non-reactive OraQuick results were diagnosed with HIV infection using EIA and Western Blot testing. Censoring these five participants the day of their final OraQuick test, the median time from the NAAT to the mid-point between the last non-reactive and first reactive oral fluid HIV test for the 40 participants evaluated was estimated to be 17.4 days (i.e., 62% of a 28 day month; interquartile range [IQR], 2.8–131.3 days).

The HIV RNA level was higher among participants receiving placebo (median = 227,863 copies/ml) at the time the NAAT was reactive than among participants receiving tenofovir (median = 5532 copies/ml; p = 0.02). We successfully amplified and sequenced HIV from 37 of the 40 HIV-infected participants evaluated; 32 (86.5%) were infected with subtype CRF01_AE, and the median plasma HIV RNA concentration was 161,878 copies/ml (IQR, 6683–541,521 copies/ml; geometric mean = 53,077 copies/ml) at the time the NAAT was positive. This was higher, but not statistically different (p = 0.15, controlling for study drug group), than the median (30,056 copies/ml; IQR, 3935–70,898 copies/ml; geometric mean = 14,840 copies/ml) of the eight participants infected with other subtypes (subtype B’ = 4, B’/CRF01_AE recombinant = 1, unable to type = 3).

**Fig 2:**
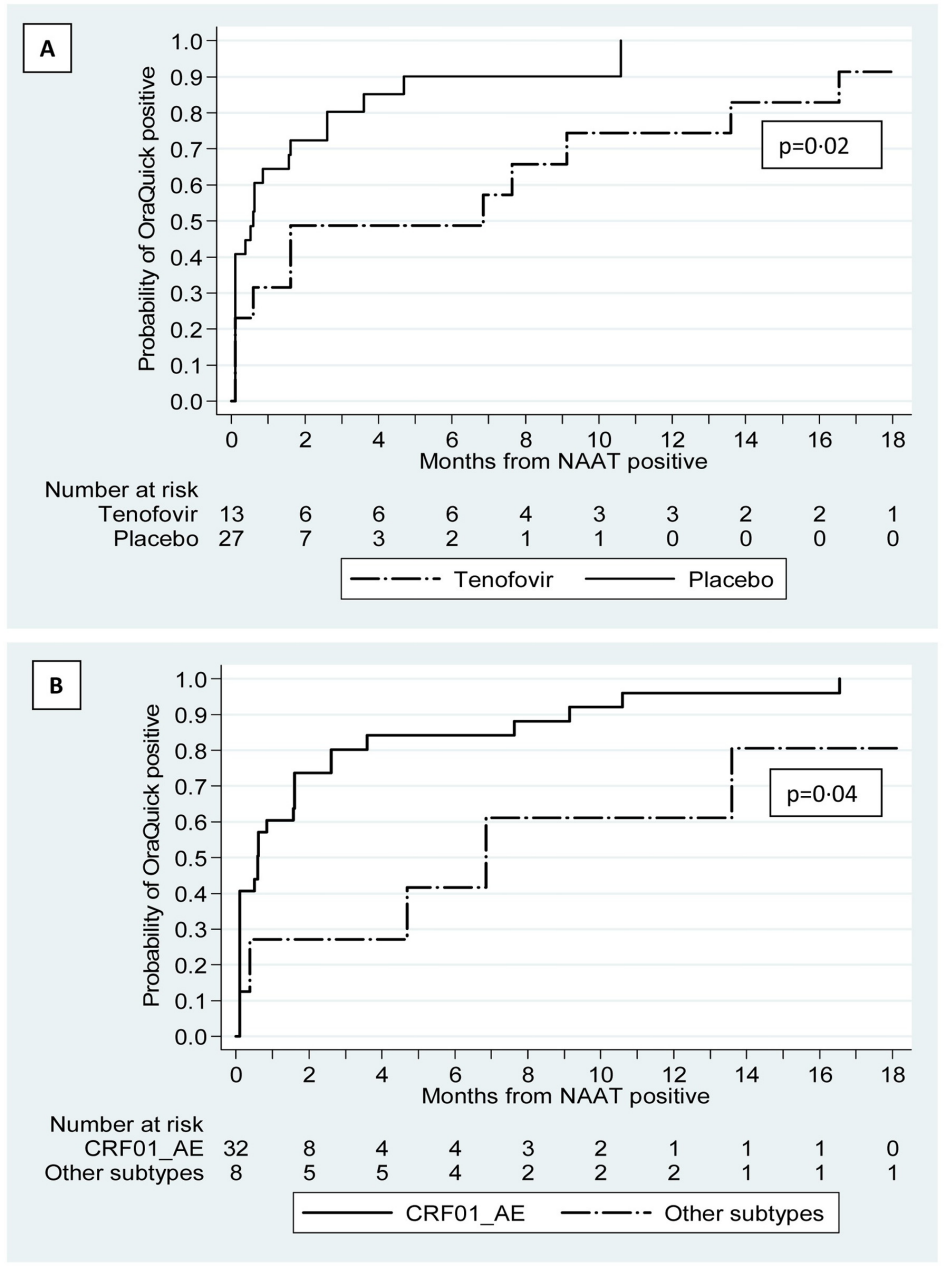
Kaplan-Meier estimates of time (28 day months) from date HIV was detected using nucleic acid amplification until the mid-point date between the last non-reactive and first reactive oral fluid HIV test. (A) By study drug group: tenofovir or placebo. (B) Controlling for study drug group, by HIV subtype: CRF01_AE or other subtypes.

The median time from the first positive NAAT to the mid-point between the last non-reactive and first reactive oral fluid HIV test for participants randomized to tenofovir was 191.8 days and for participants randomized to placebo was 16.8 days (p = 0.02). The median time for participants infected with subtype CRF01_AE was 16.8 days and for participants infected with other subtypes was 191.8 days (controlling for study drug group, p = 0.04) (Fig 2A–2B and S1 Dataset). The difference between study drug groups remained when we controlled for subtype (p = 0.05). The median time from the first positive NAAT to the oral fluid HIV test mid-point did not differ by sex (p = 0.20), age (p = 0.73), or time until the oral fluid HIV test kit expiration date (p = 0.59).

## Discussion

We successfully implemented oral fluid HIV testing in 17 drug-treatment clinics in Bangkok. The test sensitivity and specificity were consistent with performance measures reported by the manufacturer[10] and in other studies.[15,16] The positive predictive value of 97.8% in this population of people who inject drugs shows that the test is a useful screening tool, but positive results need to be confirmed with additional HIV testing. The negative predictive value was 99.9% suggesting that when used to screen for HIV infection, care providers and people who inject drugs can have confidence that a negative oral fluid HIV test result, in the absence of a recent HIV exposure, is accurate.

The oral fluid HIV test was reactive within 3 months (i.e., 84 days) of the first positive NAAT in 27 (67.5%) participants with incident HIV infection. Falsely reactive oral fluid HIV test results did not cluster by clinic, time on study, or calendar time. Consistent with a previous study showing that antiretroviral therapy can decrease the sensitivity of the oral fluid HIV test,[17] we found that participants receiving tenofovir took longer to develop a reactive test result than participants receiving placebo. We also found that participants infected with subtype CRF01_AE developed a reactive oral fluid HIV test faster than participants infected with other subtypes. Participants with CRF01_AE infection had higher, but not statistically different, plasma HIV RNA concentrations the day the NAAT was positive than participants with other subtypes. Another larger study found that those infected with CRF01_AE had significantly higher plasma RNA concentrations during the first 3 months of infection than those infected with other subtypes.[18] Higher HIV RNA levels among participants with CRF01_AE infection may speed or increase the antibody response, decreasing the time to a reactive oral fluid HIV test result.

This study has several limitations. Oral fluid specimens must be evaluated within 40 minutes and cannot be preserved; therefore, we were unable to re-test specimens to confirm the results. Oral fluid HIV tests were done monthly but blood was collected every 3 months. Thus, when NAAT testing of blood showed that HIV infection occurred before a reactive oral fluid HIV test, we had to estimate the time from HIV infection until the oral fluid test was reactive. Also, only 8 participants had positive HIV NAAT results more than 3 months before a reactive oral fluid test, limiting analysis of falsely non-reactive results.

Thailand’s Ministry of Public Health has recognized the importance of increasing HIV testing, and has issued guidance for HIV testing with same day results.[19] The OraQuick test is easy to use, does not require a blood draw, and provides a result in 20 to 40 minutes, attributes that make it a useful tool for HIV screening in settings where laboratory facilities are not available, and a rapid result and non-invasive tools are preferred. However, oral fluid-based tests are less sensitive than blood-based assays in people with early HIV infection.[16,20,21,22] During the Bangkok Tenofovir Study, staff reported non-reactive monthly oral fluid HIV test results for 8 participants for more than 3 months (84 days) after HIV infection. Thus, in a setting where repeat HIV testing will be done among people at high risk of HIV infection, for example among people receiving pre-exposure prophylaxis, a blood-based test or fourth generation test that simultaneously detects HIV-1 p24 antigen and antibody, may be a more appropriate choice.[5,23]

## Supporting Information

S1 Dataset Data used for Kaplan-Meier estimates and proportional hazards analysis. If the first positive HIV NAAT occurred the same day as the first reactive oral fluid HIV test, we estimated the time from positive NAAT to reactive oral fluid HIV test was 0 days. If the HIV NAAT was positive before the oral fluid test, we estimated the time until the oral fluid test was reactive as Time (the proportion of 28 days) from positive HIV NAAT until the mid-point date between the last non-reactive and first reactive oral fluid HIV test; Expiry 1 = more than 7 months until oral fluid HIV test kit expired, 2 = 7 months or less until expired; CRF01_AE 1 = yes, 2 = no; Sex 1 = male, 2 = female; Group 1 = tenofovir, 2 = placebo; Age 1 = 30 years or older, 2 = less than 30 years old.
(XLS)
